# Supplementary material for: Internists’ ambivalence toward their role in health counseling and promotion: A qualitative study on the internists’ beliefs and attitudes
Source: PLoS One. 2022 Sep 1;17(9):e0273848. doi: 10.1371/journal.pone.0273848 (PMC9436108; doi:10.1371/journal.pone.0273848)
Supplement: S1 File — (PDF) [file pone.0273848.s001.pdf]

Manuscript: Internists' ambivalence toward their role in health promotion: a qualitative study of their beliefs and attitudes

Supporting Information file 1. Interview topic list

Importance, benefits and topics

1. What is the **importance of promoting a healthy lifestyle for your patients**?
    - How important is a **healthy lifestyle for yourself ?**

2. Why is it **important for internists** to pay attention to lifestyle in your consultation?
    - What kind of **lifestyle topics** do you find important for your patients? Why?

Tasks, experiences and responsibility

3. How do you **discuss their lifestyle with your patients** and on what does did depend?
    - What do you do see as your task/job/role/responsibility?
    - Do you use a guideline, protocol or structure?

4. How do you **experience discussing their lifestyle** with your patients as a doctor?

5. How do you **experience a patient's ability** to change their lifestyle and maintain it?
    - what do you see as the responsibility of the patient?
    - What do you see as your responsibility?

Motivational skills, knowledge of lifestyle interventions

6. Are you familiar with **motivational interviewing**?
    - Would you take a course in motivational interviewing? Why, why not?

7. What do you know about **lifestyle interventions and where they are offered**?
    - Do you often refer? When so, where to. When not, why not?

Barriers and stimulators

8. What **stimulates or hinders** you to discuss their lifestyle with your patients?

Improvement ideas

9. What do you want to **improve?** Why?
