# Supplementary material for: Internists’ ambivalence toward their role in health counseling and promotion: A qualitative study on the internists’ beliefs and attitudes
Source: PLoS One. 2022 Sep 1;17(9):e0273848. doi: 10.1371/journal.pone.0273848 (PMC9436108; doi:10.1371/journal.pone.0273848)
Supplement: S2 File — (PDF) [file pone.0273848.s002.pdf]

Manuscript: Internists' ambivalence toward their role in health promotion: a qualitative study of their beliefs and attitudes

Supporting file 2. Internists' opinions about their role in promoting a healthy lifestyle (n=28)

	**Statements**	**strongly agree (%)**	**agree (%)**	**don't know/ not sure (%)**	**disagree (%)**	**strongly disagree(%**
1.	My patients benefit greatly if I, as a doctor, give them advice about their lifestyle	11	64	18	7	
2.	My patients benefit greatly if I, as a doctor, motivate them to change their lifestyles	14	57	21	7	
3.	Lifestyle advice is important within our team	32	57	7	4	
4.	My task is to map out their lifestyle for all my patients	18	54	18	7	4
5.	My task is to advise my patients on their lifestyle	36	57	0	7	
6.	My task is to motivate my patients to change their lifestyles	21	64	11	4	
7.	Lifestyle mapping disrupts my relationship with the patient			7	57	36
8.	My patients often fail to see that a healthy lifestyle is very important to them		11	32	50	7
9.	My patients are usually unable to change their lifestyles	7	36	25	32	
10.	Lifestyle change is the patient's responsibility	21	50	18	11	
11.	It is customary within my team to refer patients for lifestyle interventions	11	29	25	36	
12.	It is also my responsibility, as a doctor, that patients live as healthy as possible	7	54	14	25	
13.	I have too little time during my consultation to pay attention to a patient's lifestyle	14	54	4	29	
14.	When I discuss their (unhealthy) lifestyle, patients often do not appreciate it		4	25	57	14
15.	My knowledge of effective lifestyle interventions is more than sufficient		18	32	40	4
16.	My conversation skills for lifestyle counseling are more than sufficient	4	36	29	29	4
17.	When we refer them to a lifestyle intervention, patients with an unhealthy lifestyle will benefit greatly	7	14	71	7	
18.	Internists have an important role in the care network in prevention and lifestyle change of patients with complex problems	14	32	43	11	
19.	The lifestyle interventions I know of are not suitable for my patients		7	43	43	7

20. For the last ten patients at your consultation how often did you:	**0-2 times (%)**	**3-4 times (%)**	**5-6 times (%)**	**7-8 times (%)**	**9-10 times (%)**
ask them questions about their lifestyle	4	7	18	36	36
give them advice about their lifestyle	4	36	18	25	18
motivate them to change their lifestyle	4	36	39	14	7
refer them for changing their lifestyle	64	25	7		4

NB Comparing the views between subspecialties did not reveal much differences in the overall pattern except that respondents of Nephrology scored highly positive on S1,2,16 and 18 compared to other subspecialities.

Manuscript: Internists' ambivalence toward their role in health promotion: a qualitative study of their beliefs and attitudes

Description of the results of the statement-list

The importance, benefits and success of HC&P and the role of internists

Most respondents agreed with the statements: my patients benefit greatly if I, as a doctor, advise (75%) and motivate (71%) patients to change their lifestyle (S1,2). Almost all respondents (89%) agreed lifestyle advice is considered important within their team or subspeciality (S3)

Tasks and responsibilities (T&R) in HC&P

The majority of the respondents agreed with the statements that it is the task of the internist always to map out the lifestyle of patients (71%), advice patients (93%) and motivate them (85%) (S4,5,6). A minority (38%) agreed it is a habit in the team to refer patients for a lifestyle intervention (S11). Most respondents (71%) agreed that patients are responsible for changing their lifestyle, and 61% agreed internists too are responsible (S10,12).

Patient-doctor relationship, patients' ability, and internists' motivational skills

A large majority (93%) disagreed that lifestyle mapping disrupts the patient-doctor relationship (S7), and a small part (4%) agreed that patients do not appreciate a conversation about lifestyle (S14). A minority (11%) agreed that patients do not understand that a healthy lifestyle is important (S8), and 43% agreed that patients are not capable changing their lifestyle (S9). A minority (39%) agreed that they (as internists) have more than sufficient communication skills (S16)

Time, knowledge about interventions and collaboration in the care network

A majority (68%) agreed having to little time to pay attention to lifestyle during the consultation (S13). Few (18%) agreed that their knowledge of effective lifestyle interventions is more than sufficient (S15). A minority (21%) agreed (71% did not know) whether patients with an unhealthy lifestyle would benefit from a referral to a lifestyle intervention (S17). Few (7%) agreed (43% did not know) that the lifestyle interventions they know of are not suitable for their patients (S19). A minority (43%) agreed (46% was not sure) that the internist has an important (coordinating) role in the care network (S18).

The internists actual performance

The last question was: 'given the last ten patients at your out-patient clinic, how often did you: a. ask questions about lifestyle; b. give advice; c. motivate; d. refer your patient for changing their lifestyle'.

The result was that in the majority (> 6) of the last 10 cases, respondents most often asked questions about lifestyle (72%), and least motivated (21%) and referred patients (4%). Some explained the low referral scores as follows: patients come for follow-up visits; lifestyle has been previously discussed; patients have been referred already.

Conclusion

The results point to a gap between the importance the internists attach to promoting a healthy lifestyle and their actual performance.
