# Supplementary material for: Internists’ ambivalence toward their role in health counseling and promotion: A qualitative study on the internists’ beliefs and attitudes
Source: PLoS One. 2022 Sep 1;17(9):e0273848. doi: 10.1371/journal.pone.0273848 (PMC9436108; doi:10.1371/journal.pone.0273848)
Supplement: S3 File — (PDF) [file pone.0273848.s003.pdf]

Supporting Information file 3. A minimal data set of quotes

Manuscript: Internists' ambivalence toward their role in health promotion: a qualitative study of their beliefs and attitudes

This file consists of five tables with beliefs statements (propositions) and related quotes.
The belief statements correspond with the beliefs statements in the Figures 2-5 in the manuscript.
The quotes concern the minimal data set of quotes from the interview transcripts that we used to defer the belief statements.
Some quotes are used more than once to defer a belief statement since their content refers to more than one belief.

Conventions used in the interview transcripts and quotations

In the quotations we have removed backchannel words (ahs and uhms), repetitions, grammatical errors and false starts and used punctuation to improve readability. In the interview transcripts the use of backchannel words and grammatical errors etc. varied, as it reflects how the interviewees spoke.
With three dots in round brackets (…) we indicate we left out less relevant parts of the speakers expressions including repetitions and/or grammatical errors.
We used square brackets [……] for explaining remarks of the interviewer.
With a colon in square brackets [:] we indicate the removal of identifying material from the interview.
We used quotation marks within a quote when the interviewee quoted a person or themselves.
At the end of the quotes we refer only with a number to the interviewee. We did not refer to the specific sub-specialty, gender or age in order to prevent identification of the interviewee.

Table 1 **Beliefs statements and their corresponding quotes related to Fig 2**

Beliefs	Quotes[1]
**Importance of HC&P (Fig 2)**	
**Lifestyle is a major decisive factor in the biomedical outcomes and quality of life of our patients**	***Super important***. *That's in all of our guidelines (…) because* ***lifestyle is a major decisive factor in the outcomes of our patients biomedically seen and in terms of quality of life*** *(…) In essence, this applies to all patient groups, but how exactly you should fill it in will differ per patient group. That depends on the nature of the biomedical problem and the history or socio-cultural context of the patient. 38.5.1*<br>***Very important*** *because lifestyle contributes a lot to* ***the development or maintenance of chronic diseases,*** *so I do pay attention to that. I am currently doing outpatient clinics where I see a lot of vascular problems and diabetes (…) you can't see that separately from lifestyle. If someone comes up with a completely different problem that isn't related to that, then maybe I'd go into lifestyle a little less. 52.24.2*
**Obese patients with diabetes often can cure their diabetes by losing weight**<br><br>**Quitting smoking has more effect than medication**	***People who have diabetes and much overweight can often cure their diabetes by losing weight*** *and people who smoke, I often mention: 'yes, I can give you a lot of medication, but* ***quitting smoking has a much greater effect than all those medications*** *you use now.' 52.24.4*<br>*We can* ***prescribe pills, but that does not outweigh the benefits of weight loss and smoking cessation****; that is the task and responsibility of the patient himself 18.32.2*<br>*People [with hypertension or high cholesterol] who smoke, are much better off quitting smoking* ***than taking statins****.35.2.3*<br>*What encourages me to discuss this [lifestyle] is that I am convinced of its impact on the long run.* ***And I see the effects on the diabetes regulation, blood pressure(…) It really makes a difference****. And people feel much better but what hinders me is that we only have 15 minutes [for a consultation] 36.18.7*
**Success of HC&P (Fig 2)**	
**HC&P isn't very successful in most cases**	***It is a very ambiguous subject*** *ecause you can achieve quite spectacular things,* ***but with more than half of the patients it quickly reaches a dead end****. 20:14:1*
**It is difficult to influence a person's lifestyle when habits exist many years**	***In the end you reach a small group. The problem with lifestyle is that people have built up bad lifestyle habits for years*** *(..) and when they have gotten into trouble because of their bad lifestyle, we eventually come into the picture* ***and then it is very difficult to get people to change their behavior.*** *There is no recipe for lifestyle intervention. We see a patient roughly once, twice a year. Contact of a quarter of an hour.* ***Then you should not have too high expectations of what we can achieve*** *32.4.3*
**Lifestyle change often comes too late**	*Wanting to* ***change a lifestyle is a bit too late for a lot of patients****, because they come with the* ***complications of a lifestyle they have had for a long time (…)*** *the* ***damage has already been done****. 11.28.2*<br>*When people have developed dementia* ***then it's a bit late for lifestyle interventions.*** *But our patients with multimorbidity, are pre-eminently the patients for whom we try to get everything sorted out with the GP 4.17.6*
**People with a low SEP and an unhealthy lifestyle are sicker**	*It's clear that* ***some of the patients damage themselves daily due to a wrong lifestyle****. If I succeed in helping someone move forward, I occasionally see it has an effect. We see that* ***the weak, social groups [****low Socio-Economic Position* ***SEP****]* ***with an associated lifestyle are really the sicker population*** *36.18.2*
**Primary prevention is the most sensible care/approach**	***Primary prevention is the most sensible care*** *in the whole of medicine and that is largely lifestyle and that is more important than disease control 18.32.1*

[1] *The bold lines in the quotes, in particular, relate to the formulated belief statements in the left column*

**The efficiency is low but in some patients we accomplish they quit smoking or lose weight**	***The efficiency is low****, I must admit.* ***But every year I get a few people to quit smoking.*** *I think that's quite a gain. Then I am also very happy. Because in the end that is often* ***more than I can achieve with my pills and powders****. It is also often a matter of addressing the right person at the right time. (…) There are also people who very resolutely say: stop this, because I've already tried it a hundred times and I'm not going to start it again. Unmentionable. Then I will not keep coming back to it. Then I mention en passant during follow-up checks: how is smoking?* ***I'll give it another go then****. Many expect me to start talking about smoking, I can be a bit of a whiner about that. That is because I just think* ***it's very important that people stop smoking****. And that* ***we also must offer help for that****. But at the same time,* ***quitting smoking is insanely difficult. It's a very strong addiction****. I've smoked in the past, but I've never been a heavy smoker. I have also completely stopped doing it. But it is sometimes incomprehensible* ***when you see the damage people have and that they continue to smoke****. Some* ***people cannot be convinced, cannot be approached. Then I'll let it rest too.*** *32.4.74*
**Internists' role in HC&P (Fig 2)**	
**Being the acting primary physician**<br>**Having a long term patient-doctor (p-d) relationship**<br>**HC&P is part of the internists' treatment**	*If you, as an internist,* ***are the primary practitioner of a patient, then I think you should also take that aspect [lifestyle] into account****…Some patiënts we see once or twice, then I view the GP as the primary practitioner who needs to pay attention to that 20:14:2*<br>***I don't know whether internists should necessarily play a major role in this. But doctors do****. I think* ***it mainly has to do with the bond you have with your patient****- and that is why I think that* ***GPs could also do it very well,*** *they can have* ***a long-term follow-up with a patient and are therefore the right person to do that****; so, the context determines ….) who is suitable for that more than what kind of doctor you are. 10.3.3*
**Patients do *not* come to the internist for HC&P**<br>**HC&P is *not* part of the internists' treatment**	*Patients comes to me* ***for their HIV treatment, that is my direct treatment relationship; they do not come to me to quit smoking****. 11.28.4*<br>*People are treated by me for their infection and then I am not going to talk at length about their smoking and exercise****.*** *11.28.10*
Not **being the primary physician**<br>***Not*** **having a long-term patient-doctor relationship >**<br>***Not*** **having the opportunity to make a difference**<br>**GPs have a long-term patient-doctor relationship; can provide follow-up; should play a role in HC&P**	*I believe* ***it is very important****, absolutely relevant (...) but I do not practice what I preach. I don't know if it makes that much sense (...),* ***I see a patient once or twice and then I return them to the GP; then you do not have a long-term treatment relationship at all****. The assumption then is that* ***I do not have the opportunity to really make a difference****. 17.13.2*<br>*If* ***someone comes in with a [not to lifestyle related] request for help referred by the GP*** *and you see a heavily obese patient smelling at smoke,.. I will say something about it ...but whether I will do everything I can and* ***(...) would give all kinds of advice about lifestyle that should be changed? I wouldn't do that (…) that is something that belongs to the GP****. 13.6.4*
**We should not overestimate our influence and our role in HC&P**	***We overestimate our estimation of the patient and their adherence to therapy (…) and our ability to influence that (…)*** *we should be more modest (…) we have a coordinating and signaling role and need to know our boundaries. As medical specialists, we cost [society] far too much and are not really well-educated to influence that****.*** *8.7.1*<br>***I think our resources are limited (…) you shouldn't overestimate your role either,*** *you don't know the patient (…) don't practice motivational interviewing, there isn't enough time for that****. I don't know if that's our role.*** *19.31.2*

Table 2	Beliefs statements and their corresponding quotes related to Fig 3a
**Beliefs**	**Quotes**
**Assessing risk & lifestyle factors (Fig 3a)**	
**Lifestyle is a broad concept; our main task and responsibility (T&R) concerns the biomedical domain**	***It is our main task** (…) **to properly understand and carry out our biomedical task per patient group. That** is our main **responsibility** and we must take it. If we don't, no one else will. Lifestyle is a broad concept and **a lot of** the decisive factors in a specific patient cannot be covered by **the biomedical domain.** 38.5.1*
**Unlike stress, sleep, and food & drinking habits, exercise and smoking are easily assessed, and given advice upon**<br><br>**We neither have time nor expertise to go into all of that**	*When people say: I exercise for an hour in the gym five times a week, then you know [what that means]. But **I find nutrition more difficult [to appraise] because people often stay vague** about it. 47.20.3*<br>*I **don't really go into those food histories;** I send them to the dietitian. I **don't have the time** for that, and I **don't have the expertise**. **But I do have a reasonable idea of how someone can exercise and what is useful**. I feel more comfortable giving advice on that. I have the feeling that then I can achieve more too. 36.18.3*<br>*Generally, we **pay too little attention to sleep**. That is also lifestyle. Young people sometimes come to us and are always tired, but then you hear if they exercise a lot or have a relatively unhealthy lifestyle. **Short sleep or poor quality of sleep can partly be related to lifestyle**, but also to a variety of other factors. 4.17.7*
**When asking about lifestyle habits one does not get to the core**	***Inventorying how often someone exercises and what someone eats, that is almost a tick-off question**. That you know: this is **someone who does or doesn't have a healthy lifestyle** (…). So that's all very limited, **you don't get to the core**. And it disrupts [the consultation] as well. You can't get to the core of why someone …19.13.3*
**There is a lot of stress; often patients will bring this up themselves**<br><br>**Stress relates to social problems; we do not approach stress as a lifestyle related problem**	***There is of course a lot of stress**; I think that half of the consultation hours [diabetes] will be about that, but I **do not approach that as a lifestyle-related problem.** 35.2.12*<br>***I don't go into it** [stress] **too much**. **Often they come up with it themselves** (…). But if you start talking like that [about stress], your consultation **will take a long time**. So, I don't always ask directly if they have experienced stress. 21.15.3*<br>*They often come with **stress and social issues** themselves. **I only ask about it when things are not or no longer going well.** 33.12.14*<br>*We know that stress is an important lifestyle factor, but we also know that when there is stress (...) you must not emphasize their lifestyle. 38.5.13*<br>*Yes, that [stress and sleeping] is discussed very often, **but I don't see that as a lifestyle intervention**. I **see that more as a social theme** though it has to do with lifestyle. We are also very focused on the psychosocial side, it is just very much intertwined. With us you really treat the whole patient. 11.28.5*
**The government is responsible for balancing the care domain with the public health domain**<br>**In many areas of life people are limited in the choices they have**	*Nowadays a lot of people point to personal responsibility, but **there are of course many domains where people themselves have only a limited range in the choices they can make**. So those guys who work in heavy night shifts have no choice; or they don't earn enough to pay for their house. We are standing at the sideline and recognize the problems that comes from their social situation, we must draw the attention [of others] to that. But at least we should do the right thing within the healthcare domain for which we are responsible. We are dealing with a withdrawing government that does not take responsibility for the whole. **Our task as a medical profession must be to point out to the government that those different domains need to be balanced**. 38.5.1*
**Informing and advising (Fig 3a)**	
**Our main task and responsibility (T&R) is to identify**	*I think we have a **role in identifying lifestyle problems and in advising about lifestyle**, but implementing that advice, I think, should be done by the GP. 52.24.14*

**problems, raise [awareness about] the issues, explain the risks related to these problems, and may be advising about lifestyle**	*Well* ***advice, I already have a bit of doubt about that*** *because what I mainly do is outlining the problem: why it is really very important for that patient to quit smoking [in relation to their medication or other illnesses they may have (...) because everyone realizes they must quit smoking. So,* ***I see my role in raising the issue and trying to relate it to that patient.*** *10.3.10*<br>*I often explain that* ***it is my job to explain the risk of smoking*** *that it causes twice the damage with HIV and that it's my task and -not meant to be pedantic-(…) that* ***ultimately persons must make their own choices****. But [that I will help] if there's anything I can help with. 9.8.5*<br>*I always* ***explain*** *to every new patient* ***the risks of smoking****. How bad that is, just short and quite confronting (...) I try to show* ***how smoking affects their health****. 32.4.1*
**Patients know they must stop smoking, exercise more, eat healthy etc.**<br><br>**We are obliged to explicitly tell patients not to smoke otherwise it seems okay**	***Of course, telling you must not smoke; you must exercise more and you must eat healthy****; that is good to say* ***although they know this of course*** *but we as doctors must* ***tell them, that is our duty****. Subsequently the patient should be able to act upon it. 19:31:2*<br>*Then I was thinking [talking about a case]: 'Okay,* ***I need to tell people they must quit although they know it'****(…). So* ***now I do say: 'you must quit smoking'****. Maybe people just need that. 35.2.9*<br>*That is important.* ***Because when the doctor doesn't say anything about it, then the patient will not bring it up either****. Some people say: '****the doctor doesn't say anything about my smoking, apparently it's okay'****.* ***I just must say it****. 34.2.1*<br>*You can't have the patient coming home, saying: 'the doctor, fortunately, has said nothing about smoking. 8.7.7*
**It is our task and responsibility (T&R) to inform patients well to make a well-informed choice**<br><br>**Patients make their own choices; if they do not want to change, it is within their right/own responsibility**	*I always* ***try to tell them what the effect is, so that they are informed****. Then I say: 'now you are at least informed and* ***you can do whatever you want*** *with that information'. 21.15.6*<br>*I think* ***it is important for patients to inform them about this [risks of an unhealthy lifestyle****]. But I think* ***it is the patient's responsibility to deal with it. It is my job to name it and to give advice****.49.19.2*<br>*If(…)* ***it is a well-considered choice, because I actually think that is my task****, and a person does not feel like it now, then I am completely at peace with that (…) As a doctor you think what a waste of your kidneys. But if someone does that deliberately, then I don't see the doctor as an authoritarian figure who says: this is how it should be done but* ***more as a source of information that informs patients. When they say, 'I want to continue to smoke anyway', I don't think I have not done my job well*** *49.19.5*<br>*The question is what you see as your responsibility.* ***People have autonomy over their own lives. I can advise and educate them about that and if they want, I can refer them or give advice myself*** *and sometimes people benefit from that.* ***But there are also plenty of people who know very well that they have a bad lifestyle and don't want to change it.*** *And someone may think that too, i****t's not that they don't know, It's a choice they make.****11.28.2*<br>***[My task is] to inform them well, but if people do not want to, it is their own good right****.* *HIV increases e.g., the risk of cardiovascular disease. So smoking is not a smart thing to do but* ***patients come to me for HIV treatment*** *and if they clearly indicate 'I've heard you and I'm not prepared to make any concessions and I understand what the consequences are'* ***then I am not going to push it any harder.*** *11.28.4*

**Table 3**	**Beliefs statements and their corresponding quotes related to Fig 3b**
**Beliefs**	**Quotes**
**Motivating (Fig 3b)**	
**Motivating patients costs time and energy, and is not very effective**<br>**We should not have high expectations of what we can achieve in the short time of a consultation**<br><br>**You need to assess whether it makes sense to make an effort to motivate patients**<br>**Sometimes you do invest**<br>**It is frustrating when patients are not willing to do the right thing and just expect a pill from the doctor**	*I can sometimes approach people very direct, stating this is good for you (…) but after fifteen minutes they are walking out of the door. At some point, they come back and nothing has changed.* ***Sometimes you see the impossibility of your own interventions or intentions (…).*** *There are some things* ***you really don't get to with 3*****x** ***fifteen minutes a year****. 33.12.15*<br>*We see that patient* ***roughly once or twice a year****. Contact of a quarter of an hour.* ***Then you should not have too high expectations of what we can achieve****. 32.4.3*<br>***A lot of people are not like that*** *(...)* ***or do not immediately adjust their behavior****. (…)* ***Some people cannot be convinced, cannot be approached. Then I'll let it rest too.*** *32.4.74*<br>***With one patient I put more energy into it than with another (...). I estimate whether it makes sense to do so****. With some I think I see an opening* ***and apparently feel that it makes sense*** *for this patient to start it. And when my impression is: '****it's still carrying water to the sea' then I will not put much energy into it.*** *17.13.7*<br>***How far do we have to go as doctors****? As an internist I can indicate in the letter to the GP that the patient is overweight and [advice] to send the patient for a [lifestyle intervention, but if the patient does not do anything with it himself (…) -I think that is very important, you can't leave everything to the doctor- (...).****It is a doctor's task to motivate patients but if the patient in the end says: 'I do not care at all', then it makes no sense.*** *13.6.18*<br>***It's very frustrating*** *when people keep coming to us for follow-up and doing things that aren't right for them and* ***you feel like you can't find an opening*** *to talk about it. Some people* ***place the problem outside themselves****; then the doctor should do something about it.* ***Then they expect a pill*** *for high cholesterol or high blood pressure and then they are not going to do anything about it for it themselves.* ***Then it's more like a supermarket, y****ou only come to get something. 25.1.14*
**Patients are more motivated after a life event**<br>**Patients must be highly motivated, or we will waste time and effort**<br>**Some we need to approach gently, others in a direct, confronting way.**<br>**Some patients we have given up**	*For most people* ***a life changing event [heart attack, stroke, surgery] is the time to change things*** *(…) then I say: 'it has been a warning and now is the time to think about your life'. 18.32.7*<br>*Of course, there is not one best approach (…) You really need to see how you get through to people and with some you should talk not so much as a doctor but as a person. I happen to wear a white coat and people better listen to that (….)* ***Some need a tough approach and some we have given up*** *and I have the idea that I* ***can estimate in advance for whom it is totally pointless to try something like this [motivating to lose weight] and it is a waste of money and effort****. One has to* ***be incredibly motivated [as a patient];*** *otherwise one can stop:* ***motivation is number one****. 18.32.17*<br>***It all depends on whether the patient is motivated*** *to lifestyle change. 52.24.6*
**Patients must have the (cognitive) ability to understand**<br>**Sometimes against better judgment, we keep encouraging self-management**	*Self-management is very high on our list (...). I try to put it in the hands of the patient as much as possible. Maybe* ***sometimes against our better judgment, we will keep doing it (…)****.* ***You always have a group of people who don't understand it; they don't see it, cannot process it or are cognitively not yet ready for it****. Then it gets difficult. 44.21.4/6*
**Patients sometimes only need to be listened to/understood**<br>**It doesn't always**	***Sometimes people need a time-out and a listening ear****. Then it's about establishing a relationship and showing understanding for a miserable situation and then you shouldn't go on about it and wanting to achieve something (…)* ***You are not always capable to do something about it.*** *8.7.3*

**belong to your ability to do something about it**	
**Patients are responsible**<br><br>**It is not our task to invest in motivating patients**	*A large proportion of my patients with functional complaints has a lifestyle-related problem. (…) I usually don't have much to treat with regard to the somatic content, so I have some time (…) to talk about these kinds of [lifestyle] things (…). I also largely leave* ***the responsibility with the patient*** *(…) You must steer them a bit (…) But* ***I don't think it's my job to hold a motivational talk every three weeks.*** *14.9.11*<br>*We have 45 minutes for a new patient (..)* ***then I have the time to address lifestyle and that motivates,*** *and sometimes I have the feeling that you really have meant something to them,* ***even though they actually have no somatic problem.*** *14.9.15*
**Supporting & Referring (Fig 3b)**	
**You expect patients to take the initiative to go to the GP if we refer them back for Lifestyle guidance**<br><><br>**We must take immediate action to arrange for patients going to a program; otherwise, they will likely cancel or postpone it**	*Even though I write it in my letter,* ***I still think patient should go to the GP themselves for [lifestyle guidance].*** *I do not think that the GP should call that patient. 49.19.16.*<br>*If you feel that people are willing to invest in it themselves and want to do something about it, I will see how I can help them. Possibly refer them if you cannot do that yourself (…)* ***But in principle I think it is up to the patient to deal with that****. 49.19.2I*<br>***I do not take any action myself*** *(...)* ***So I refer someone to a GP or tell them that they can go to the GP for that****. I often put it in the letter, but* ***that's all I do about it****. 10.3.6*<br>***The stop-smoking out-patient clinic is no longer there. I regret that, that would have helped****. (...) Now I must refer people to the GP again.* ***Then the question is whether they will go.*** *Maybe it's better through the GP (...)* ***But they still must take that step first to go to that doctor.*** *34.10 19*<br>***Patients benefitted greatly from our stop-smoking out-patient clinic****,* ***then I would have called immediately*** *(…) because once you have discussed it,* ***immediate action must be taken otherwise it will be [postponed****] (...). Sometimes I call [the GP] -I recently saw a young lady with a very high BMI (...), her overweight was the biggest problem of all her complaints- then I call the GP (…): please do you want to actively pursue this, because the greatest gain can be made with a lifestyle change. That always works very well (…) then it will be picked up better. 52.24.11*<br>*I have also* ***tried to persuade people to go to the stop-smoking out-patient clinic****, or to go via the GP (…) but then my experience is that* ***people cancel that appointment anyway*** *because they are just not motivated. 21.15.6*<br>*Only diabetic patients (...) can be referred to a dietician here. Other patients are dependent for [lifestyle] interventions on primary car****e*** *(…) Only very occasionally* ***I refer them to the dietician her****e,* ***if I think it would be such a shame if we lose the momentum****. 29.16.6*
**Patients with an unhealthy lifestyle are not very complex from a biomedical perspective**<br>**Internists are overqualified; not reimbursed and do not have the knowledge and motivational skills for lifestyle interventions**<br>**Carrying out lifestyle advice belongs to the GP and is often**	***I don't think these patients are so complex from a biomedical perspective****. I think we have a role in identifying lifestyle problems and in advising about lifestyle, but in implementing that advice I think this should be done through the GP practice (…).* ***A nurse is often appointed for this****. 52.24.14*<br>***From a biomedical point of view, I do not find these patients very complex****. They are often people who are in a particular [social] situation or are stuck in a specific pattern, where* ***lifestyle, among other things, plays a prominent role; in that sense they are very complex patients*** *17.13.4*<br>***Regarding the counselling process, I don't think you need an internist for that. We are a kind of overqualified*** *(...). It* ***is also about [reimbursement]*** *and I also think that we are not competent because* ***I do not have the knowledge of all those lifestyle interventions*** *and have* ***not yet been trained in motivational interviewing*** *10.3.14*<br>***We cost [society] too much to counsel for smoking cessation or lifestyle interventions****. If we want to do research on lifestyle interventions, we might have to work with the general practitioners department.* ***There is no reason for doing that all here****. 8.7.11*

**delegated to a practice nurse**	
**Lifestyle issues are overall the same in all patient groups**<br><br>**There is no need to do [protocolized] lifestyle guidance in a hospital**	*I do think there are specific problems within specific patient populations.* ***But when you're talking about smoking, exercising, and eating healthy, and maintaining a healthy weight, yes that won't be any different for any population****, I guess.10.3.31*<br>***You do not need to support that [Lifestyle advice] in a hospital****.* ***A complex patient does not make your lifestyle advice different; they are basically always the same,*** *except in the case of specific dietary changes or so. 52.24.13*<br>*You should be able* ***to delegate what is protocolized*** *-if you are overweight* ***you should eat less and exercise more and that is not something very complicated to do.*** *8.7.7*
**HC&P should take place in patients' social environment**<br><br>**The travel distance to the hospital is a big problem for many patients**<br><br>**GPs can see them more frequently**<br><br>**GPs are responsible and should play a central role**	*I think it's possible [lifestyle interventions in primary care] because I would very much like to get rid of the [HIV] patients' stigma and I think our patients want that too. So, I don't think you should treat them differently from other patients because they aren't different patients. Maybe a different risk factor here and there, but that is the same for every other patient group.* ***But when it comes to a lifestyle intervention that you must follow up on a long-term and regular basis, I think the travel distance is really a big problem****. 10.3.15*<br>*Especially* ***if you are in a university hospital and you are so far away from the patient's neighborhood you should not take everything away from that environment****. I think that* ***the GP should always keep a central role****.19.31.4*<br>***The region is too big, you can't see them all here*** *(...).* ***People also give up when they must travel for lifestyle advice****. Regular contact seems to help (…) with video calls or whatever that keeps people motivated. But some (…) will also find it very patronizing if you focus on that. 32.4.10*<br>***The majority just lives too far away and I don't know how much they would like it if they had to come a few times a week to the hospital*** *and pay parking fees every time.(…). Then I tell them the GP may likely know a good dietician or physiotherapy practice in the area. 34.10.10*<br>***For someone with type*** **2 diabetes*****, it is not always practical to come here every week for a dietitian. They often come from far away****. If it's* ***possible, you should look for help closer to home*** *(…) I also refer [not diabetic] patients to physical therapy, in primary care, leaving it with the GP.* ***And some GPs have affinity with HC&P and some will let it go completely.*** *29.6.17*<br>*Actually,* ***I now mainly refer people to the GP*** *(…) For example, regarding smoking cessation pointing out that there are several options, there are aids and that the GP can help. (…) Sometimes I prescribe something,* ***but you don't act on it, don't have weekly contact*** *with the patient (…)* ***as a practice nurse*** *in general practice would do. There is one* ***person ultimately responsible for a patient*** *and I think* ***that is very often the GP and then the practice nurse is the one who should do it*** *(...). To organize it here, we would need a supporting staff, which we don't have. 19.31.3*
**Our HIV or renal patients do not go to their GP for problems GPs should treat**	*Our HIV* ***patients hardly ever visit their GP****. Because for every complaint they go to the GP with,* ***the GP very quickly says: 'go to the hospital because we never know for sure whether it is HIV-related or not'****, it could be due to side effects of the [HIV]medication. So, in that sense, they are very much focused on us. 10.3.15*<br>*Many* ***patients see us as GP*** *(...) also* ***because of the stigma****. They know that* ***we know their main diseases well****.* ***GPs also confirm this****. They very often say: 'just ask your internist'(…).* ***If you find [lifestyle] a very important part, then it is logical that you also take the lead in this) (...).The disadvantage is that we have a large region and it is not suitable for everyone [here]****.(…) The best thing would be that it's organized in health care centers in their neighborhood and that we give the incentive* ***and it is picked up well in their environment. Not all problems are HIV-related, so, let the GP be in charge*** *for those 7.11.7*<br>*Unfortunately, the GP also says* ***if the patient has a GP problem: 'you are going to the hospital anyway; have them have a look at it'****. They also often think* ***that is too difficult****.*

	That does not mean that every doctor does it that way, certainly not, but a lot of them do. 44.21.7<br>*Or people who say on the doorstep: 'I also suffer from (...)'. So, a**ll GP questions are asked to me**. 34.10.7*
**Our academic patients need integrated care**<br>**We should offer lifestyle guidance in the hospital, now it's only for a selected group**<br>**Patients often cannot afford lifestyle guidance**	*I can send someone separately to the dietician, I can send someone separately to the gym, etc.(…)* ***but that is not an integral treatment*** *as with the diabetes rehabilitation program [in our hospital] with even a psychologist involved 18.32.12*<br>***I want to treat them here****. Sometimes I feel ashamed that I cannot refer…****we are the highest address and then I must tell people just look at that website and go to a gym and that is not affordable. (...)*** *I* ***can prescribe them pills but I cannot offer them good [lifestyle] guidance) in our own hospital and I am just ashamed of that****. 18:32:14*<br>***Only ten physiotherapy consultations are reimbursed*** *... and then it ends 18:32:16*<br>***The problem is what next?*** *If you persuade someone, are we going to send them all to the GP? I also have patients who say, '****I have already been to the*** *GP then it costs more****. I want to go to the stop-smoking out-patient clinic but it no longer exists.*** *7.11.6*
**GPs have the same problems as we do: many competing tasks and limited time and will delegate it to practice nurses**	*[Do you refer the GP?] Not really, I don't know what they can do;* ***I think GPs have the same problem as me; they do not have time (…) they need to delegate it too****. 18.32.18*<br>*Frankly,* ***that is something a GP also could do very well*** *[listening to /understanding patients] they know the [family] system [they know what has happened],* ***but they only have five or seven minutes*** *(…) and now they have* ***practice nurses for this and for that (…).*** *Would you like to be a GP in that situation? I don't know 8.7.3*<br>*I often write (in the letter to the GP]: 'I would like you to take up the guidance of the patient for irritable bowel syndrome (IBS)' (...)* ***I will never hear about that again.*** *I think it's disappointing [not to know what happens at the GP] because enough patients already have a diagnosis of IBS, and then I tell them things about IBS and they say they ha****ve never heard that before (…) I*** *don't know* ***whether the GP really has time for that [educating patients]; they only have ten minutes for a consultation.*** *14.9.13*<br>*Quite a lot of people managed to quit smoking with help [of our stop-smoking out-patient clinic] but* ***that kind of guidance has now also been delegated to the GP who doesn't have time to give the right guidance because they already have so much on their plate.*** *54.30.8*
**We need to monitor the progress ourselves**<br><br>**E.g., for HIV- or renal patients, the expertise of GPs and other primary healthcare professionals is hardly sufficient**	*But those [cognitively less capable] people also need more exercise. I would like to arrange that nearby [in the hospital] because I don't like handing this over to the GP. Then I must ask the GP to arrange that locally and hope it will be done. 44.21.6*<br>*And I miss the consultation moments with physiotherapist or other health care professionals: how are things going? I want to hear their feedback. 44;21;14*<br>*I recently heard from a patient that she was very dissatisfied with the dietician elsewhere because the dietician knew nothing about kidney transplantation. She had given advice that she should not have given, especially when a patient uses immune suppressive drugs. Clearly, not a success. 47.20.3*<br>*I prefer to have patients with our own dieticians, who know exactly what to do with those (insulin) pumps because if people must lose weight, the insulin dose has to be reduced. They can put people on a low-carb diet and are good at motivating people and to keep it up 34.10:8.*<br>*The dietitian provides very thorough counseling at the diabetes out-patient clinic. 15.10.20*
**We must convey as an organization how important a healthy lifestyle is**	*It's madness.* ***If you emphasize this [a healthy lifestyle] as an organization and it stops*** *right there. Of course offering smoking cessation here would be desirable for our population. It's a bit* ***questionable how far our responsibility goes****. But I can imagine that* ***the hospital advocates more supply in the region for exercising in leisure time*** *(…).* ***To convey the signal that you care****. 36.18.13*

Table 4 Beliefs statements and their corresponding quotes related to Fig 4

Beliefs	Quotes
**Beliefs about the patient-doctor-relationship (Fig 4)**	
**You need a long-term treatment relationship to make a difference**<br><br>**One needs time and frequent conversations**	*But I do not practice what I preach. I don't know if it makes that much sense (..).* ***I see that patient once or twice and then I return them to the GP; then you do not have a long-term treatment relationship at all****. The assumption then is that I* ***do not have the opportunity to really make a difference*** *17.13.2*<br>***You need to have eye to eye contact from time to time****, which is really more effective than via an app, mail or telephone. If I start coaching people myself (…) I occasionally see people [mainly diabetes patients] every other week at the out-patient clinic when I think much can be gained, and yes* ***that takes a lot of time (…) I think it really makes sense if I continue my efforts as a doctor and call or see them for a while, and e.g. let them e-mail me for example every day*** *(...). You do see a relapse though, but you may have gained at least 5 years where they did not need insulin 18.32.19*<br>***Regular contact seems to help*** *(…) with video calls or whatever to keep people motivated. But some people will also find it very patronizing if you count on that. 32.4.10*<br>*With a few patients I have a more* ***long-term relationship****. Then I can sit and talk for half an hour (…). Such a bond to develop takes time (…) and can be useful. 14.9.11*
**To start a conversation about lifestyle, one needs trust and an opening; otherwise, it only will be a sermon**<br><br>**We should not preach or say you must**<br><br>**We should avoid blaming patients; they may feel stigmatized**	*With one patient I put more energy into it than with another (…). .I estimate whether it makes sense to do so. With some I think* ***I see an opening and apparently feel that it makes sense*** *for this patient to start it. And when my impression is that it still will be carrying 'water to the sea', then I will not put much energy into it. 17.13.7*<br>*If you want to have this kind of conversations,* ***you need to trust*** *each other to a certain extent, and I will wait for that but not endlessly 44.21.4*<br>*It all* ***depends on whether the patient is motivated*** *to make a lifestyle change. What I do now is that I go into that [lifestyle topics] once, very clearly,* ***trying to avoid preaching****, so I try motivational conversations. I don't bring it up until later in the conversation,* ***when you've already have built a bit of a bond with the patient,*** *and you know what someone's life looks like (..) That is of course important for knowing their lifestyle (…).* ***So, there must be an opening to have such a conversation. Otherwise, it will be a sermon, and it will miss the mark.*** *52.24.6*<br>*I start with the question: 'are you okay with us talking about it'.* ***Stating 'you must do this and you must do that', I haven't done that for years(****…).First provide the patient the opportunity to open the door to a discussion about this (…) then ask a positive question, then, the patient will come up with the reasons why he wants to change. 8.7.2*<br>*If everyone* ***keeps saying stop smoking and pointing that finger****, they may feel* ***stigmatized****. Maybe he has made quite a few attempts already, but it just didn't work. 48.22.8.*
**One needs to establish a bond; otherwise, patients will not tell you what bothers them**<br><br>**We should not judge people so they can openly speak about their lifestyle**	***You try to establish a kind of contact with patients (****…) and then you can or cannot raise certain issues in that contact (...) and of course you try to help them when you notice they have moved in a certain direction (..) and it is very nice when they succeed [I give an example]. That patient did it himself, of course we have supported him and coached him (…)* ***you talk to people regularly and that might help.*** *19.31.6*<br>***You must have a certain bond with the patient so that if things are not going well,*** *with liquor, drugs, or one's job,* ***they will let you know****. That's why you try to get a picture of the social situation.* ***Ultimately, you want a good relationship with every patient****, but I think we* ***might invest extra [in the relationship] to obtain that information****. I also try* ***to decide together with the patient how and when to take medication.*** *Since adherence to therapy is so important, you also touch upon those other [lifestyle] things a bit. 9.8.4*<br>*As doctors we have the responsibility* ***not to judge people*** *and* ***to make sure they can openly speak about their lifestyle*** *how much they drink and/or smoke (…) and why. 4.17.34*

**Delicate topics (overweight, using drugs during sex) may stress the patient-doctor relationship**	*What hinders me is that your* ***treatment relationship with the patient might get into trouble then****. The emotion that the patient expresses at such a moment. You might notice that e.g.,* ***weight is a very delicate subject for someone*** *and with smoking that it might be 'mind your own business'. 20.14.9*<br>*People just don't know what they are eating.* ***When you address what food they eat, you are more likely to stress the relationship than talking e.g. about exercise.*** *33.12.4*<br>***Sometimes I find obesity difficult to discuss with young women because it is so confrontational*** *(...) with young people. Maybe I fill it in myself, that I think it is important to have good looks when you are young, whether or not yet in a relationship (...). So,* ***that [touching upon other subjects] sometimes gets in the way.*** *52.54.10*<br>*I'm really not going to tell them every time* ***they shouldn't use drugs while having sex because then I have a disrupted my doctor-patient relationship.*** *11.28.2*
**After renal transplantation, lifestyle is easily negotiable/patients find it normal/not annoying/expect it**	***In the transplant clinic*** *there are* ***many people who bring up whether they can do something about it themselves, because a transplant is a positive life event****.* ***Those patients really want to do everything they can to keep that kidney in (****…).I have an approach (…) where you also learn from their feedback (...). So in principle* ***lifestyle change is easy negotiable within the context of regular transplant care****. 38.5.11*<br>*In my opinion,* ***patients consider the discussion of these topics as paying serious attention to them****.* ***I don't experience that as a disruption in the relation****. I cannot rule out the possibility that they are occasionally defensive, but no, I do not experience it that way 36.18.6*<br>***I also think that most people perceive that [questions about lifestyle] as normal****.(…).If people find it annoying, they say so too (…).Then I always try: 'but you know why it is important?' (…). In some cases, it just does not work and I regret that very much 47.20.7*<br>***I have never experienced that patients find it annoying*** *(…).* ***People also expect you to ask that.*** *[People may be defensive when they don't want to talk about quitting smoking?] Yes, but then it's good that we have talked about that once. I****t never feels uncomfortable to me****. 49.19.14*
**Beliefs about the patients' ability (Fig 4)**	
**Everybody knows that smoking and being overweight is bad for your health**	***Everyone knows that smoking is bad*** *(…). That is the single most effective measure to get people healthier. 38.5.9*<br>***There are very few people who don't know that smoking is bad, that obesity is bad. Everyone really knows that;*** *how they should achieve a change is less clear. With smoking it is simple: quitting is the only solution, you don't have to be an intellectual for that; what healthy nutrition is, is already a bit more complicated.18.32.3*
**Most people understand it very well but do not want to change; are not motivated at all; it is a choice**	*They [patients] also indicate that* ***they know it is a problem****. Some also say they've tried it for a long time, but don't have any tools.* ***And some of them laugh it away and just go on talking while ignoring it****. I may stop then,* ***but I will come back to that later****. 44.21.5*<br>*There are also plenty* ***of people who know very well that they have a bad lifestyle*** *and do* ***not want to change anything about it****. And they are allowed to think that. Yes. (…). I'm not going to tell them every time* ***they shouldn't use drugs while having sex because then I have a disrupted my doctor-patient relationship (...) It's not that they don't know. It is a choice they make****. 11.28.2*
**It's useless trying to motivate some patients to change their lifestyle**	***Some just do not want to****; they will continue to smoke and eat unhealthy food; they accept that they will live shorter lives (...).* ***They should decide that for themselves****. 18.32.3*<br>*Some people need a tough approach and some* ***we have given up*** *and I have the idea that I can estimate in advance* ***for whom it is totally pointless to try something like this [motivating to lose weight]*** *and it is a waste of money and effort 18.32.17*
**Some patients cannot be saved; cannot understand/ be motivated/**	***There remains a patient group that cannot be improved****. And then you can send them to [a program] again, but that is (…)* ***not very promising*** *(…). But it happens (…) simply because you have no other options. Finally, you must accept it. 20.14.12*

**are addicted**<br><br><br><br>**When they are obese, have a low SEP and limited resources, and poor insight into their illness, you first need to address the social problems**	***With HIV you have a group of people who are more likely to be addicted*** *and you can keep harping on it, but at a certain point* ***they will no longer come to you with their problems****.* ***With a diabetes patient it is essentially not very different****, and of course it is for a reason they have diabetes type 2;* ***they are addiction sensitive people****. So, we can emphasize that very much; an****d it works for ten percent of the people but not for the majority.*** *It is an ethical discussion, it has to do with autonomy and what someone needs from you.11.28.11*<br>***Addiction care has been an eye-opener for me in the past year****; (...)* ***[in addiction care] all unhealthy lifestyle habits are labeled as addictive behavior: sitting addiction*** *etc. 8.7.1*<br>*People with a complex illness (…) who articulate well and have some degree of self-determination will also more easily participate in and benefit from lifestyle programs;* ***those are not the patients [with a*** *low Socio-Economic Position (SEP)]* ***of whom you think i****n advance* ***it will not work*** *(..). There [in a low SEP area]* ***you have a lot of people with overweight, and limited resources and insight into illness****. That is a reality (…). That is a challenge. You first must* ***solve a number of other social things*** *(...) After [a program of] 2 years,* ***you see that a large number of people relapse****. 19.31.11*
**Obese diabetes patients are seen as fat, stupid people who eat too much and exercise too little**<br>**Older, obese people with a metabolic disorder will not lose weight anymore. When we act normal about it, we may prevent they become frustrated and gain weight**	*Compared to e.g., patients with cancer,* ***obese patients with diabetes are seen as fat and stupid people who eat too much and exercise too little.*** *29.16.11*<br>*I can get very angry when the cardiologist tells* ***a 66-year-old with a metabolic disorder****,* ***you need to lose fifteen*** *kilos.* ***That is simply not going to work anymore*** *(…)* ***That will frustrate them and ensure that afterwards they just gain weight again****. So maybe one day I won't put them on the scale. (…) People always think that they are getting fatter. Then I say: 'No, you don't get fatter (….) You're getting older'.* ***That helps so much. Just, acting normal about it****. 29.16.2*
**Patients vary a lot; with some you feel stuck if they can't stop smoking**<br><br><br><br>**Some do understand but cannot address it now or are not yet at the stage to change**<br><br><br><br><br><br><br>**We should address it the next time**	*I am also very sorry to see if people (…) really cannot stop smoking, that they just don't see it. But some see it, but can't do anything about it. I'll just keep saying it, but sometimes you feel stuck (…) You are looking for an opening. (...) It varies a lot, some say it will not work in advance (…) and still others don't feel like it. 21.15.6*<br>*You get varying responses [when discussing a referral to a dietitian]. Sometimes people say: I don't really think that's necessary yet. Or: I'll think about it myself first. 47.20.3*<br>*Sometimes patients continue to hold off [lifestyle talk]. I regret that very much. 47.20.7*<br>*I turn my monitor over, even at my [out-patient] clinic, then we go through the results together. And I feel that it works for most patients (…). But you will always have a group of people who don't understand it, don't see it, they cannot process it, or are cognitively not yet ready. Then it gets difficult. 44.21.4/6*<br>*No, [patients] generally [do] not [change their lifestyle], that is a small minority; The last patient I saw (…) didn't want to, continued to smoke, drink, and were overweight. I confronted him in the hard way. He has changed course now, suddenly wanting.18.32.3*<br>*And sometimes you notice that people are not motivated at all to change their lifestyle, do not find it important or do not feel that they can control it themselves. Sometimes I let it go, then I think: this is something to come back to later, there is no space for discussion now. Usually, I have the feeling that it does lead to a good conversation. Sometimes people say, I've been trying to quit smoking for so long, but I just can't. Some are very convinced: 'because of my work I live very irregularly and that cannot be changed'. 52.24.6*
**Beliefs about internists' motivational skills (Fig 4)**	

**It would be good to have these skills as doctors for specific patient groups >**<br><br>**So, you can use them when needed or problems persist**<br><br>**We don't have time to use these skills**<br><br>**We do not get reimbursed if we spend more time**<br><br>**It will never be the core of my consultation**<br><br>**May be better to organize it with a nurse**	Ultimately, **you wonder if you could do it more practically and effectively**. So that's why I also find that [motivational interviewing] course interesting. **There are some things you really don't get around with 3x fifteen minutes a year**. I can sometimes approach people very direct, saying this is good for you (...) **but after fifteen minutes they are walking out of the door. At some point they come back and nothing has changed**. S**ometimes you see the impossibility of your own interventions or intentions.(…) Then I think you must approach that differently**. 33.12.15<br>With some people I think **it really doesn't make sense** and that I should let it go, **but there is a category of patients who may benefit if I had more time and was better trained in it [motivating]**. 49.19.6<br>I would participate [in a training], **I think it is very nice and very important to understand the patient's motives**; that also helps you a little and thus the patient. If you can set that up for selected groups such as transplant patients and look at what the goals are, and ensure that you do something that will last, **then you will achieve something**. 19.31.12<br>But sometimes you feel stuck. **Those are the situations for which I would like to have some better conversational skills**. Motivational conversation, I am curious if that helps e.g., regarding food, it would be nice to try it. You are looking for an opening.21.15.6<br>Whether I should do it in a different way, I don't know**. I wonder if you should not take an intermediate step to refer somebody to someone with experience in motivational interviewing and then maybe a referral**. I don't have the time to have such a long conversation. **The question is also how useful it is to spend time on it, if you don't have the skills** (…) it is hard to say what technique helps to quit smoking. Some patients want to try it with [nicotine patches or gum] others need a motivational conversation. 21.15.7<br>And how they will stop, **we really have too little knowledge (...) to really guide them and** (…)too little **time**. I can of course make more appointments, but I have just explained. 10.3.10<br>**I wouldn't use that [motivational Interviewing] for every consultation, but when the problem persists, you can make time for** it somewhere in a 15-minute consultation. 9.14.17<br>If 70-year-olds have lived a certain way all their lives, and it always went well, they say: 'why should I now suddenly need to change' (…) it takes time to get people to this point. I think we generally have enough expertise to do this, except for complex patients 13.6.20<br>Well, **I would sign up for it (training motivational interviewing],** because **there will always be opportunities** (…) **but it will never be the core of my consultation** that I tell people their lifestyle (...) But if it is the case then you have a skill to approach that in an adequate way, **but I would not use motivational interviewing by default with all my patients**. 11.28.13<br>I**t would be good if doctors were also trained [in motivational interviewing]. I**n case you think you may need it, **then you can do that optimally**. But I also understand that **it will cost more money to give the doctor 15 minutes more consultation time**. That it **might be better to cluster it with a nurse**. 49.19.7<br>To discuss lifestyle **time is above all I think the most important**. 49.19.14
**You need to have an affinity with motivating patients and a patient group that will benefit of it**<br><br>**For therapy-resistant obese diabetics, new medication will more likely make the real difference**	**Motivational interviewing is something we can try to apply quite easily**. It will not suit everyone, **you must have a real affinity for it and also have a chronic patient group where it will really matter** and that you see more often and where you really keep control as an internist. 20.14.22<br>We mainly **see people who have had one or two therapy failures**. **Or they have multimorbidity and problems** (…) **of which we conclude that it is a lifestyle-related problem**. **If we tackle obesity and diabetes type 2**, the rest [morbidities] should also improve.(…) Then we **need the tools that allow us to deliver a little mor**e. I also think that **pharmacotherapy** - semaglutide and liraglutide or other agents - **has added value**. That will really be a game- changer in the next three to five years. **Then we can really make a difference just for those who turn out to be therapy-resistant**. 33.12.18

**It is hard to estimate whether training is useful**	*I have **never had any training** in **this kind of [motivational] interviewing**. **I do not know it. I cannot rule out the possibility that it may be useful, but I have too little insight into it to be able to judge it at all**. I am open to that in principle. I would **consider training, but it depends on the time investment** 36.18.14*
**Maybe a training will be useful**	***That [motivational interviewing] could of course be of value (…). I do not know**. You know what you know and you do it your way. But of course, it may be that **if you are going to a course you will learn something from it; it certainly could. I am always open to that**. 47.20.8*<br>*I have been looking online once, to see if I could find some information about [motivational interviewing]. But I have followed no official training. **Think it would be nice if it was organized within the internal medicine department** 9.8.7*<br>*A year and a half ago there was a motivational interviewing course that we had registered for. **It was cancelled because there was not enough enthusiasm for it**. But that was eight evenings or so. Yes, **quite** a lot, but **if you want to learn it, you must put in the time,** I assume. 10:3:8*
**Training should not take too much time**	*I wanted to do that, but it has never happened. **There was a course that was very intensive - ten times an afternoon or so, that is too much, it is not possible to do that extra**. I'd like to do motivational interviewing for a day or so. It's on my wish list. 52.24.8*<br>*I think it might be useful and **I want to take a course in that, but how much time does that take? Where do I find the time to do this?** 34.10.16*
**We should not invest in these skills if we cannot offer follow-up visits**	*I don't know whether I can really make a difference, but I can at least initiate it to make a difference in an entire care chain. And I **don't think I have enough tools to do that properly yet. I think I could learn (…) a certain way of communicating (…) to set things into motion**. But on the other hand **I do feel (...) I only want to invest in that when I can offer follow-up (...) otherwise I really get a very unsatisfactory feeling**.17:13:13*<br>*I also think that **we are not qualified because I do not have the knowledge of all those lifestyle interventions out there and I have not yet been trained in motivational interviewing** or any other strategy that you can best use for this.10.3.14*
**I am doing pretty well; I do not need such training**	***That's fine with me.** I have practiced a lot, have been doing this all my life, 38.5.11*<br>*I don't really like to pat m**yself** on the chest, but **I think I have a pretty good command of that: entering into a conversation with a patient. Personally, not so much need**. **I do not feel I miss something on that front**. Look, if I regularly have a lot of friction with the patient or the idea the patient does not understand me or does not pick it up, then I would indeed say yes. 44.21.15*<br>***I think I'm doing pretty well for each patient**. But maybe there are more people who think that and that it is not true. But no, I am usually very open and fairly direct in just making it negotiable and asking why it does not work. 48.22.**9***

**Table 5**	**Beliefs statements and their corresponding quotes related to Fig 5**
**Beliefs**	**Quotes**
**Beliefs about (lack of) time (Fig 5)**	
**Everything has to do with the amount of time you have available. So, you set priorities/make choices**<br><br>**What is the most urgent gets the most attention**<br><br>**Priority lies with a patient's primary need for help**<br><br>**Lack of time limits us to go into lifestyle issues <> Except in the case of Medically Unexplained Symptoms**	***Everything has to do with the time that you have available and the priorities you set,*** *and the priority for me lies with* ***the patient's primary need for help*** *11.28.18*<br>*When people come for checkups more often and have many different issues,* ***you can of course not a****ddress* ***everything when you have only 10 minutes****.* ***Then you make a choice*** *and save the other issues for the next consultation.* ***What is most urgent gets the most attention at that moment****. Sometimes I decide that, sometimes the patient and sometimes we decide together. 25.1.14*<br>*(…) but everyone sees the benefit of it.* ***Unfortunately, we don't have the time to do it ourselves*** *and I do think it matters if the doctor says so. 18.32.12*<br>*What hinders me is that* ***we only have 15 minutes****. Often there are* ***many other things involved*** *as well. There's just* ***not much time to go into it in depth****. But that much time is often not necessary in my opinion; better talk about it for 30 seconds than not at all.36.18.7*<br>*I think* ***it's important enough to make time for it, so I'll just run out of time a bit.*** *24.10. 5*<br>*You* ***just don't have the time to bring it [lifestyle] up every time****. But if there's any reason for it, I'll start about it. 33.12.2.*<br>*A large proportion of my patients with functional complaints has a lifestyle-related problem (…). I usually don't have much to treat with regard to the somatic content, so I have some time (…) to talk about these kind of [lifestyle] things; (…) It is* ***still only have a quarter of an hour and that is of course nothing for motivational conversations****. 14.9.11*<br>***We have 45 minutes for a new patient*** *(..) And that you spent 45 minutes is already an eye-opener for them. They are examined, they can tell their story, (…)* ***then I have the time for this and that motivates,*** *and sometimes I have the feeling that you really have meant something to them,* ***even though they have nothing somatically.*** *14.9.15*
**We need to do/discuss a lot in a diabetes consultation**<br><br>**We only have 10-15 min. for everything**<br><br>**There is no time left to discuss lifestyle issues or stress**<br><br>**Time always runs out; that's very annoying**	***I have fifteen minutes for the check-up. This is of course disastrous in case of diabetes patients****. First the blood sugars, then blood pressure has to be measured and if things are not going well, a foot examination must be done. And then taking a look at the lab. And certainly, if people have a pump and sensors, just printing out blood sugar values takes up* ***a lot of time****.* ***It always runs out. So that's very annoying****. Then you are already lagging while the patient thinks the doctor has all the time (…).* ***You'll never catch up unless someone else drops out (…).*** *Or people who say on the doorstep: I also suffer from (…). So,* ***all GP questions are also asked to me. I also have too little time for lifestyle topics****. 34.10.7*<br>*With diabetes you usually discuss risk factors (…)* ***that is a bit of an overloaded consultation*** *in which* ***you actually need to discuss too much, then you split that up a bit and it is not always discussed. There is no time for this because you must check 100 things during the annual visit****. 20.14.4*<br>*I'm trying to get some more clarity on that (sleep and problems), so that you might be able to give some advice.* ***But I have fifteen minutes for everything****. Bringing in the patient, asking how things are going, taking measurements, placing orders, you name it.* ***Those 15 minutes will be gone in no time****. I cannot cover everything with questionnaires and in-depth interviews. It does not work. 32.4.84*<br>*But if you start* ***talking about that [stress], your consultation will run out of time****. So, I don't always ask directly if they have experienced stress. But I often hear it, people often bring it up. 21.15.3*<br>*They often come with* ***stress and social issues*** *themselves.* ***I only ask about it when things are not or no longer going well*** *(..) sometimes you touch upon something, and sometimes you don't(...)* ***If everything goes well, you can sometimes finish in five or ten minutes (...)*** *And sometimes* ***you take longer because there are indeed psychological problems****. And*

**Time lacks to find out e.g., which physical therapy practice is nearby**	*stress at work, or at home, those are really things that deregulate their blood sugar. 33.12.14.*<br>***And if you proceed to ask [about diet], the fifteen minutes are over in no time**. There is always a discrepancy between a patient's body weight and how much they say they eat. I leave that to the dietician 33.12.4*<br>*If you know which patient has a good chance of success and which patient does not, then that [motivational interviewing] is of course a good tool; **the only thing that is difficult is to do all that within your consultations because the patient also comes for something else [medically]. T**hat is difficult, **you actually need all your time for that.** 35.2.27*<br>***This is beyond my job** description **and certainly the time I have per patient. So, I can just give t**he **advice** to **go to the GP**. **Then it takes much less time than if I must figure out which physiotherapy practice is nearby**; that is impossible. 34.10.12*
**You should see it in perspective**<br><br>**We spend hours on administration; must do everything ourselves**<br><br>**We must click too much in the EHR**<br><br>**We spend a dis-proportionate amount of time on improper tasks**	***And you must see everything in context** - now I'm going to whine - that we as doctors must **do more and more administration**, that in fact everything that needs taken care of is put on our plate (…). Everyone is so overwhelmed [with administrative work] that they are just fed up with everything; that's really a problem. 18.32.21*<br>***You must make choices**. I have twenty minutes for a follow-up visit and forty minutes for a new patient. There are many things to tick off if you work according to the protocols. And you're also **endlessly clicking in Epic**. At a consultation hour you try to get the atmosphere right, and then you make choices. 8.7.4.*<br>*The challenge is to maintain a personal conversation despite the laborious and **time-consuming electronic health record.** (…) **We spend a disproportionate amount of time on improper tasks.** After a morning in the out-patient clinic you are sometimes still busy for two or three hours (..) But what is protocolized, we should try to delegate. 8.7.7*<br>*We have **so much to do in Epic**, that's my resistance. I now type very briefly, short notes what I find relevant. But nobody uses those fill-out exercises for history-taking and physical examination, **it's just too much click work**. I'm faithfully going through smoking, alcohol, drugs and allergies as a flow list in Epic, but if lifestyle is added to that, I'm afraid you're just filling out items [without going into their relevance]; so that would be my resistance. See, smoking is short: that is yes and no (...), but if you really want to do it well and fill it out [lifestyle topics in detail] **then you are of course busy for some time, right?** Height and weight, blood pressure, measuring, **I must do all myself.** Some clinics have arranged for the secretaries to do that, as well as blood pressure and pulse, **but I do everything myself**.*<br>*I would be fine with patients providing information on basic lifestyle topics in the EHR for example by completing a questionnaire about diet, exercise, alcohol, smoking. 14.9.17*<br>*It happens that someone [when entering the room] is already a bit agitated, because the blood pressure measurement did not go well; or because there was a new digital registration post they didn't understand (…) **Then I will be talking for 5 minutes** [to calm the patient] **before I've done anything. Good preparation [**of the consultation] **is much more important** than for example a lifestyle dashboard. Also, **that the medication list is checked and properly stored in the EHR in advance**. We are increasingly moving towards that. 49.19.18*
**Knowledge of interventions & Collaboration in the care network (Fig 5)**	
**We lack referral options within the hospital: i.e., supporting staff or integrated/ specific/trusted interventions or programs. These are only available for**	***For a selected group you do have tools**. The diabetes rehabilitation program [.] for example has the total package, but **not everyone is eligible for this, not even people who are seriously overweight** without having diabetes, while they also might benefit from it. That is one of the few referrals I really believe in because I see that (…) people are approached integrally, and I would love to be able to tick lifestyle coach [in the EHR]; a lot of people would qualify for that. The stupid thing is it all [lifestyle interventions] costs money outside the hospital. 18.32.12*<br>*I can prescribe medication, **but I cannot offer them good [lifestyle] guidance in our own hospital** and I am just ashamed of that. 18.32.14*

**specific patient groups**	*What good is my therapeutic armamentarium? That's only referral to a dietitian for people who want it.* ***Those lifestyle coaches that's for all of us yet a bit unclear****.* ***I happen to know it's reimbursed****. I must get experience first****. Really know it (…) that it has proven itself*** *(...).*<br>*I often leave it to primary care, the general practitioner. S****ome GP's have an affinity with it and some let it go.*** *If so, at the next visit we must come up with an alternative. And very sometimes I also refer them to the dietitian here, when I think it is* ***such a shame if we lose this momentum*** *(…). Our dieticians are only for diabetic patients with [insulin] pumps, who need multiple injection therapy and by no means for all. 29.16.6*<br>*I would like it if* ***primary care would also be much more enthusiastic to take this task [lifestyle guidance] on****, accessible and close to [people's] home. And if people find it difficult to leave their house, maybe at their home. Connecting more with people, see what fits someone and what someone likes. 29.16.7*<br>*You must* ***have the tools [lifestyle programs/support] in your own hands*** *and be able to offer it to the people. But that is often lacking. 44.21.1*
**We have no overview, no knowledge of the interventions or programs offered in Primary Care (PC)**<br><br><br><br><br>**We have no insight into the expertise of PC dietitians or physical therapists for specific patient groups**	*I do* ***not have the knowledge of all those lifestyle interventions*** *[in Primary Care] and have not yet been trained in motivational interviewing or any other strategy that you can best use for this. 10.3.14*<br>*I* ***have no idea how to get in touch with a primary care physical therapist****,* ***I don't have any tools at all to refer****. We used to have specific referral notes, but they no longer exist (...)* ***We also don't have a lot of insight into what is reimbursed****. I'm not very educated on that..(…)*<br>*It would indeed* ***be good to have an overview of the interventions in the reference area for exercise, nutrition and smoking****. Ideally, you should be able to refer as easy as possible. That with one click in your patient file in the EHR and a referral note etc. comes out 36.18.1*<br>*And if you refer to the GP, because that is actually the person who has to deal with it as the current system is organized now, th****en it varies a lot per GP practice*** *what kind of staff they have (…). I especially noticed that* ***the quality of dietitians varies*** *(…) So I really* ***don't have much insight into how well it will go afterwards****. I know they have certain programs. 21.15.7*<br>*Recently* ***I heard from a patient that she was very dissatisfied with the dietician elsewhere*** *because* ***the dietician knew nothing about kidney transplantation.*** *She had given advice that she should not have given, especially when a patient uses immune suppressive drugs****.*** *Clearly, not a success. 47.20.3*<br>***I prefer to have patients cared for by our own dieticians****, who know exactly what to do with those pumps because if people must lose weight, the insulin dose has to be reduced. They can put people on a low-carb diet and are good at motivating people to continue 34.10.8*<br>***I miss very much that I cannot refer the patients with diabetes to the physical therapist here****. It would be very useful if they discuss with the patient what the most convenient place is in the area. I* ***have absolutely no overview of physiotherapy practices in the area, who does what and who is good at it****. And,* ***I don't think it's my job to figure that out.*** *34.10.10*<br>*The dietitian at the diabetes outpatient clinic here provides intense supervision.* ***Physical therapy is not in the picture****, but it would be good to offer that also or to combine it 15.10.20*<br>***I have never referred anyone to a physical therapist****, but I have referred to an exercise program(…) elsewhere. I know that* ***there are a lot of [providers]*** *and I let my patients google for providers in their own neighborhood or I do it myself, and next my patient and I have a look together what is offered. 35.2.29*

**We have no insight into the content, quality and reimbursement of lifestyle interventions in PC**<br><br>**PC dietitians or physical therapists do not inform us about progress and results**<br><br>**After referring, we do not get feedback from GP's in return**<br><br>**Our region is big. We must deal with many GPs; we do not have short communication lines/ know them anymore**<br><br>When you search **you will learn where disease-specific programs for e.g., fibromyalgia are offered**	***If you can have the blood pressure reading the GP has measured, I find that useful (…). Even if the patient says that the GP said it was fine****, I am not really sure whether the blood pressure reading was fine in line with their treatment goal. 9.8.11*<br>***Currently, I don't know what that GP has discussed with the patient [about lifestyle topics].*** *It is of course a bit irritating for patients that we might start talking about it again, while they had a similar conversation with the GP yesterday and have said they no longer felt inclined to change it. (...) If you see that is the patient is doing well, then you also can give a compliment 9.8.12*<br>*I also think it is partly a GP's responsibility, so I often write [in the letter to the GP]: 'I would like you to take up the guidance of the patient for irritable bowel syndrome (IBS)'.* ***Next the GP has to find a dietician and prescribe medication. (...) I will never hear about that again.*** *I think it's disappointing [not to know what happens at the GP], because enough patients already have a diagnosis of IBS, and then I tell them things about IBS and they say:* ***'I've never heard that before'.*** *There is also a recall bias, certainly in this patient group, but I don't know* ***whether the GP really can find time for that; they only have ten minutes for a consultation.*** *14.9.13*<br>*I miss the stop-smoking outpatient clinic. I was always satisfied with that. I had quite a few patients who went there if they were motivated. It didn't always work, but often it did.* ***You could also always see exactly what had been discussed with the patient, how things were going*** *and when the next contact was scheduled, so you could relate to that. Maybe they had very good reasons for that [closing the clinic] (..). I understand that you need to make a strategic choices [at the hospital level] and say that it all should be done in primary care.* ***But the advantage of the stop-smoking outpatient clinic here was that we also got something in return. We never get anything in return*** *(...). Now we supposedly have a lifestyle guide to refer someone to. But that is much less intensive.* ***This is of less use to us than the intensive support by the stop-smoking outpatient clinic.*** *9.8.1*<br>***And yes a program here, [in the hospital] would be nice (****…) that we can say: 'go there they will help you to start' and then after two or three visits it is best to continue [under supervision] in primary care. (…) Over the years I* ***have learned where providers are who are able to offer something more to patients [****with chronic fatigue or fibromyalgia]****.*** *I refer them there,* ***but I never hear back if it helps.*** *14.9.15*
**Investing in a regional care network focused on lifestyle is a good option**<br>**<>**<br>**Investing in a care network in a low SEP area costs a lot and does not yield much; it might be better to spend the money on making the population more prosperous**	***I think it would be desirable*** *to concretize [follow-up] together with e.g., the GPs:* ***that we create a care pathway over the walls of this hospital*** *(…). I could imagine that would be my priority. 17.13.9*<br>***I would be willing to spend time on that, so we could create something like the heart network in our region (…) I think that's a challenge (****...) but one mainly focused on lifestyle. And then I would also like to think about this with several other relevant [stakeholders] in order to make a plan for it, and to involve also patients. 17.13.168-170*<br>*You also must think carefully about what you ultimately want to achieve in the long-term and for whom [****in the context of a low SEP area****] (…) How can you get people to exercise? That you offer a program. There will always be some people who will participate but many will not.* ***It's still a big gray, underdeveloped field and then you can't control the quality*** *(…)* ***I don't know if you should [set up a network]. I'm afraid that if we set it up, it will ultimately end up with nothing****. That's why I'm a bit negative about it. It does cost a lot of money that you also could spend to make [low SEP regions] more prosperous. 19.31.11*
