# Supplementary material for: Internists’ ambivalence toward their role in health counseling and promotion: A qualitative study on the internists’ beliefs and attitudes
Source: PLoS One. 2022 Sep 1;17(9):e0273848. doi: 10.1371/journal.pone.0273848 (PMC9436108; doi:10.1371/journal.pone.0273848)
Supplement: Attachment — Submitted filename: Response to reviewers comments April 24.docx [file pone.0273848.s004.docx]

**Response to the comments of the reviewers by the author**

**Concerning** PLOS ONE Decision: PONE-D-22-01465
Internists’ ambivalent understandings of their role in health counseling and promotion

A. **Response to the comments of Reviewer #1**

Reviewer #1: This is really a nice topic to be published. It handles the beliefs and attitudes of internists about their role as Health Advocates in health counseling and promotion. In a qualitative method of research. Efforts of researchers are clear and make excellent logic.
The following suggestions would be needed for better publication.

1) **Comment
   There should be a clear notification in the title and within the abstract that this is a qualitative study.** The model was developed based on qualitative methods. **In the keywords, the authors mentioned qualitative analysis, but this is not enough to let the reader know that this research is qualitative research.**

**Response**
We added the following subtitle: “a qualitative study on the internists’ beliefs and attitudes**”** **(See line 3**). In the abstract we specified we used qualitative methods and that the study was part of a Participatory Action Research project (**See line 30-34).**

2) **Comment related to Standards for Reporting Qualitative Research**
   ‘’The authors in **lines 166 – 167** confirm that they adhere to the Standards for Reporting Qualitative Research (O'Brien et al., 2014)’’
   **Please note that the citation style is not matching with the citation style throughout the paper.** Please correct it.
   Authors in content fulfill most requirement Standards for Reporting Qualitative Research, **however, the heading – subheading especially for the method section should be re-written to match the SRQR and make it easier for the reader to flow.**
   <https://journals.lww.com/academicmedicine/_layouts/15/oaks.journals/ImageView.aspx?k=academicmedicine:2014:09000:00021&i=T1-21&year=2014&issue=09000&article=00021&type=Fulltext>

**Response**
First, we corrected the citation style for O’Brien et al 2014 **(See line 182).**
Second, we have moved the theoretical framework section under Methodology and changed its name in: Qualitative approach and research paradigm (**see line 98**). We further changed:
Study design in: Data collection methods **(See line 128)**
Participants in: Sampling strategy (**See line 152**)
Ethical approval in: Ethical approval pertaining to human subjects (**See line 176**)

3) **Comment: Line 115 – 116**
   ‘’we formulated the following research question:
   what are the internists’ beliefs and attitudes about their role in HC&P, and how does this affect their understanding of this role?’’
   **‘’ Figure 6. Internists’ beliefs and attitudes regarding their role in Health Counseling and Promotion (HC&P)’’**
   Comment,
   **Actually, the figure illustrated a very nice sum-up of the belief.** **If the authors can add a sum up of the attitudes mentioned in previous figures, then this figure will be comprehensive and provide the full answer to the research question.**

   **Response**
   We added a sum-up of the ambivalent attitudes in Fig.6 (**See Figure 6**).
4) **Text lines: 114 - 115**
   ‘’We based the interviews with the internists on topics identified in research on internists’ strategies to reach productive interactions (48) and on constructs related to theories of health behavior that informed research on promoting a healthy lifestyle in primary care (18,19,21,49,50).’’
   **Comment**
   While the reference for internists’ strategies to reach productive interaction was specific and clear, unlike the references for constructs related to theories of health behavior. if the authors can be more specific that would be better. Or the author can list main theories for examples theory of Reasoned Action or Theory of Planned Behavior etc.

**Response**

We have rewritten the content of the subsection: “Data collection methods” to be more specific about the theories that informed the design of the interviews conducted as part of the PAR- project and about how this study relates to the PAR project **(See lines 128-150).**

5) **Text lines: 120 – 124**
   PAR project was mentioned first time in the method section, with a non-English reference number 47. I think it is worthy to have some lines about the PAR project in the introduction, to support the background for a better introduction.
    **Response**
   We have changed the last lines of the introduction to provide some background about the Participative Action Research (PAR) project and added an English reference **(See lines 90-95**).
6) Text lines 131 – 134
   Please provide the questions that were used in the interviews, lines 131 – 134 mentioned the topics only without the questions used. Were the questions and discussions in English?

**Response**
We added S1. Appendix I: the interview topic list with the interview questions translated in English. The interviews were held in Dutch. (**See line 145 and S1. Appendix I**)
We also added S2. Appendix II, results of the statements list, because the interview questions related to the items in this statement list (**See line 146 and S2. Appendix II**)

B. **Response to reviewer #2**

**Reviewer #2**: The authors interviewed with 28 internists of six different subspecialties from one academic medical center in the Netherlands to explore their role in Health Advocates in health counseling and promotion (HC&P). Finally, they found these internists were less ambivalent about their role in HC&P, when they viewed HC&P as a part of their treatment and believed patients were motivated.
This is a secondary analysis of the qualitative data from a participative action research (PAR) project. The reviewer worried this kind of secondary analysis would be too much purposely factitious lead to much bias.

**Response**

Presenting the study as a secondary analyse has caused doubts about potential bias. On second thought we think that the following may have contributed to the idea of bias: using the term secondary analysis without a clear notification that it concerned a qualitative study and without explaining that this qualitative study concerned a specific research question to the interview data gathered in the PAR project.
To avoid further misunderstandings we have rewritten the “Data collection methods” section (lines 126-148) with the purpose to be more specific about how this qualitative study/analysis relates to the interviews as part of the PAR-project (see also response 4 to Reviewer 1). We first describe the aim and design of the interview studies as first step in the PAR- project. Second, we make clear that this scientific article, subsequently, focuses on the internists’ understanding of their role in health counseling and promotion by an in-depth analysis of their beliefs and attitudes in the interview data gathered within the PAR project, and based on our theoretical framework and a more specific research question.
Finally, we did not use the term secondary analysis anymore because the term as such does not seem to contribute to a better understanding of the qualitative research process involved.
